# Endoscopic removal of sinonasal hemangioma using vessel sealing device without tumor embolization

**DOI:** 10.1002/ccr3.8148

**Published:** 2023-11-02

**Authors:** Hong Jin Kim, Ye Hwan Lee, Seung Hoon Lee, Min Young Seo

**Affiliations:** ^1^ Department of Otorhinolaryngology – Head and Neck Surgery, Division of Rhinology and Sleep Medicine Korea University College of Medicine, Korea University Ansan Hospital Ansan‐si South Korea

**Keywords:** endoscopy, hemangioma, vascular closure devices

## Abstract

When massive bleeding is anticipated during endoscopic sinonasal tumor removal, a vessel sealing device is useful for successful tumor removal.

## Graphical abstract

This case report describes the successful removal of a large benign sinonasal vascular tumor using the bipolar vessel sealing system (LigaSureTM) without preoperative tumor embolization failed. The surgeon successfully removed the vascular origin tumor without embolization using LigasureTM device.

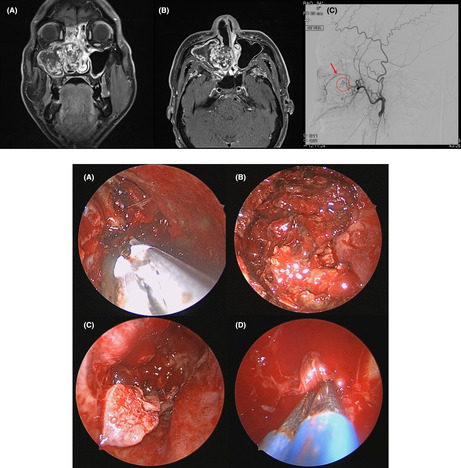

## 1 INTRODUCTION

Endoscopic removal of benign sinonasal vascular tumors is challenging for endoscopic surgeons. Profound bleeding during surgery interrupts the clear endoscopic view of the surgical field and results in incomplete removal of the tumor, which causes recurrence.^1^ Therefore, tumor embolization using angiography 1 or 2 days before surgery is a crucial preoperative treatment for successful surgery.^2^ However, tumor embolization may not be possible in some cases. In such cases, extensive bleeding is inevitable during surgery, resulting in conversion to an open approach or incomplete tumor removal. In the present case, we share the experience of successfully removing a large benign vascular tumor using LigaSure™ (Medtronic, Minneapolis, MN, USA) when preoperative tumor embolization failed.

## 2 CASE REPORT

**FIGURE 1:**
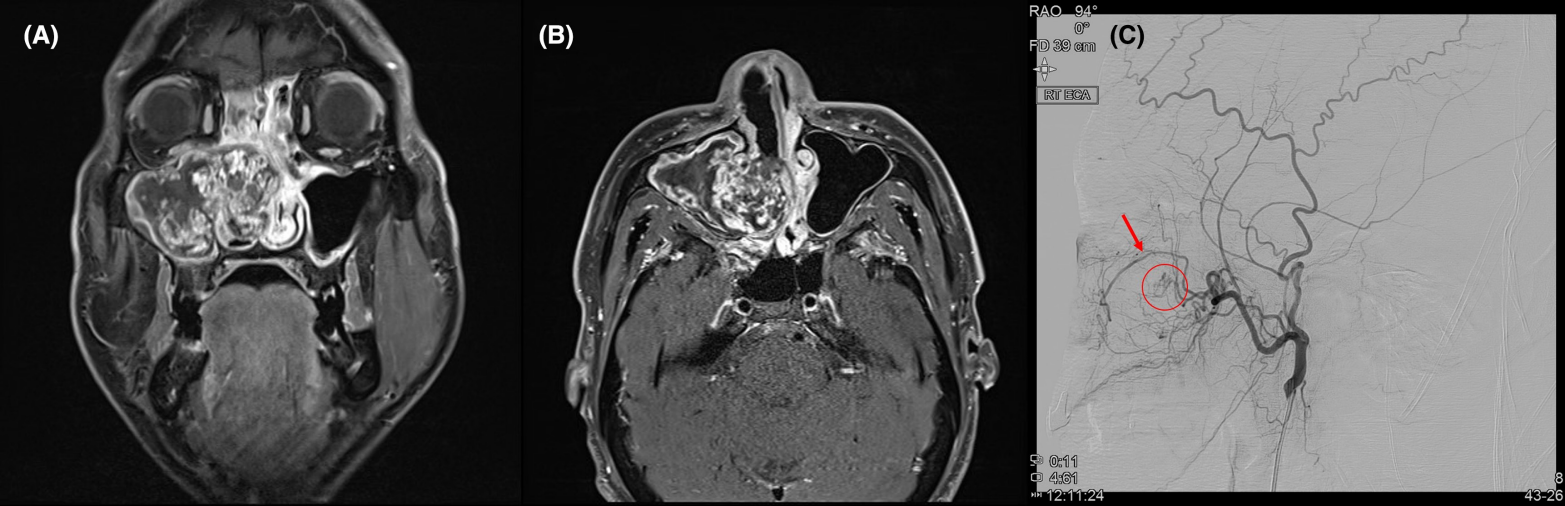
T1‐weighted gadolinium enhanced coronal and axial magnetic resonance images (A) and (B), and angiographic finding (C) of the tumor.

**FIGURE 2:**
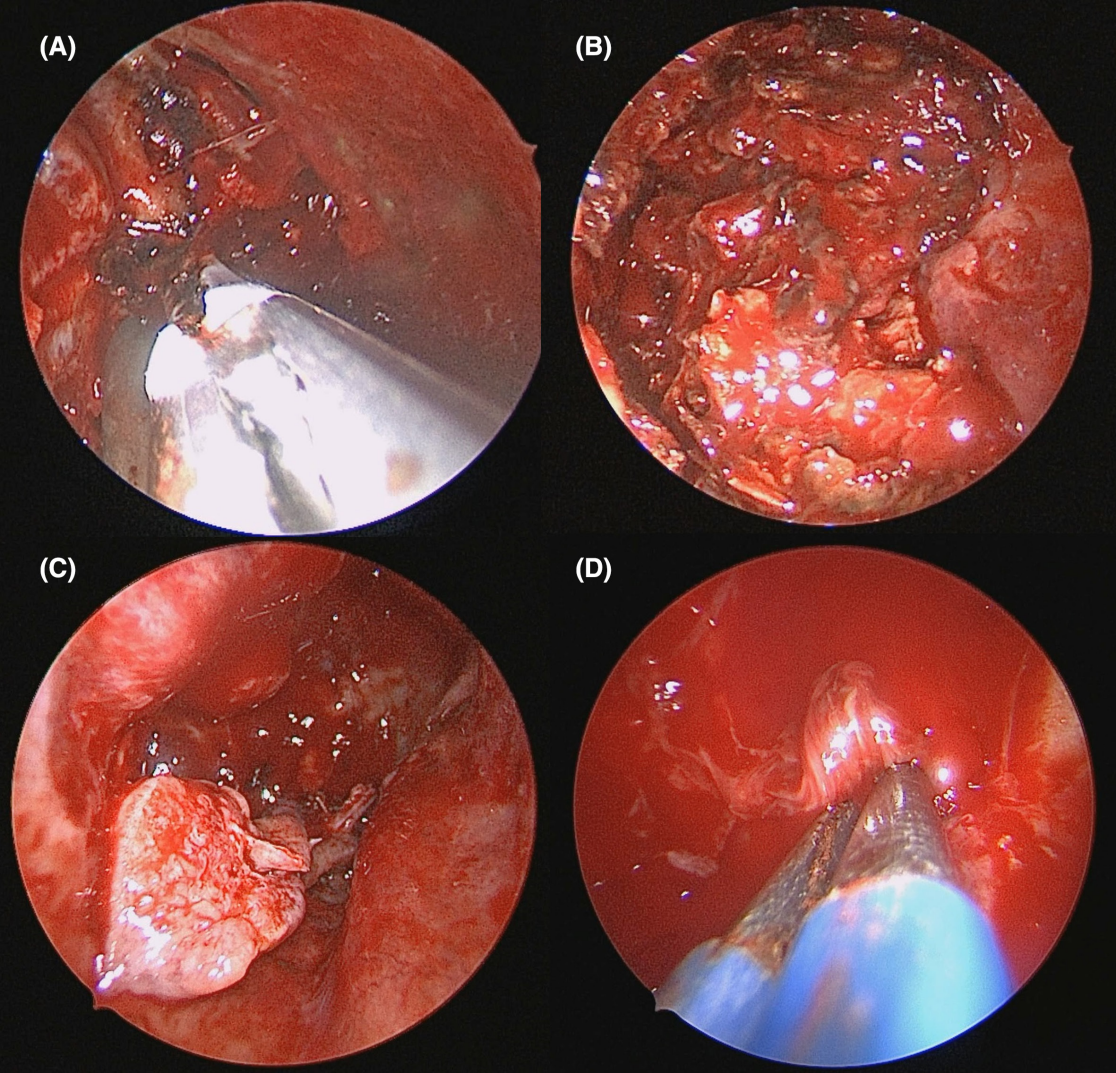
Tumor removal using LigaSure™ system (A), removal of first part of tumor using piecemeal resection (B), and endoscopic finding revealing that the entire tumor was removed (C), and vessel pedicle was bipolar cauterized (D).

A 44‐year‐old man presented to our clinic with bilateral nasal obstruction. Endoscopic examination revealed polypoid lesions and irregular surface tumorous lesions in the right nasal cavity, and the nasal septum had deviated to the left side. We performed a punch biopsy of the tumorous lesion using Blakesley forceps without imaging workup, and profound bleeding was observed. To control the bleeding, we applied epinephrine several times at the bleeding site using cotton pledgets, which was unsuccessful. Thereafter, we attempted bipolar cauterization of the bleeding focus; however, the bleeding was not arrested. After an hour‐long attempt to control bleeding, we packed a large amount of Vaseline gauze into the right nasal cavity and admitted the patient. After admission, magnetic resonance imaging (MRI) and computed tomography (CT) revealed a mottled enhanced mass (approximately 5.5 cm) in the right maxillary and ethmoid sinuses. This expansile mass caused remodeling of the medial and posterior bony walls of the maxillary sinus and the inferior orbital wall (Figure 1A). Therefore, the radiologist suggested several possible diagnostic results, such as hemangioma, hemangiopericytoma, and organizing hematoma. Accordingly, surgical removal was planned after tumor embolization using angiography 2 days before surgery. Angiographic results revealed that the tumor was supplied by the internal maxillary artery, and flow to the ophthalmic artery was also observed; therefore, embolization could not be performed because of the possibility of blindness (Figure 1B). Thus, we attempted to remove the tumor without embolization using the medial maxillectomy approach with the inferior turbinate swing technique.^3^ At the initiation of the surgery, we removed all packed materials and attempted to cauterize the surface of the tumor using a bipolar system. However, profound tumor bleeding persisted, and the surgical endoscopic view was obstructed. Therefore, we could not perform en bloc resection and opted to remove the tumor by piecemeal resection. We used the LigaSure™ vessel sealing system to remove the tumor while minimizing bleeding (Figure 2). We successfully removed all tumors with an estimated blood loss of less than 300 cc. The patient's vital signs were stable throughout surgery and during the postoperative admission period. All packed materials were removed 2 days postoperatively, and the patient was successfully discharged on the same day.

## 3 DISCUSSION

Through the development of endoscopic video systems and instruments, the treatment of sinonasal, lateral, and anterior skull base lesions using an endoscopic approach has rapidly developed.^4^ The surgeon can avoid morbidities and complications through the endoscopic approach compared with the traditional open approach.^5^ Furthermore, in the outcome of olfactory neuroblastoma treatment, the endoscopic approach may even show better disease‐free and overall survival rates than the traditional open approach.^6^ Therefore, the successful removal of sinonasal tumors through the endoscopic approach is very important if endoscopic access is possible.

Preoperative tumor embolization followed by endoscopic removal is a standardized technique for sinonasal vascular tumor resection.^2^ Without embolization, the surgeon may fail to completely resect the tumor and recurrence may occur, and sometimes, the operation may be stopped because of extensive bleeding.^1^ In some cases, massive bleeding can lead to death. Therefore, the endoscopic removal of vascular tumors is challenging for surgeons.

LigaSure™ is a bipolar electrosurgical instrument that permanently fuses vessels up to 7 mm, which provides a combination of pressure and energy to create vessel fusion.^7^ To date, there has been only one reported case of vascular tumor removal using LigaSure™ in the otorhinolaryngologic field. In this case, the authors removed a hemangioma <2 cm in size located on the tongue.^8^ We regard our case as more complicated because complete resection of the large tumor was required using an endoscopic system. Using a vessel sealing device, the entire tumor was successfully removed without embolization. Therefore, we suggest that if preoperative embolization fails, it would be better for patients and surgeons to attempt endoscopic removal using advanced instruments before considering an open approach.

## 4 CONCLUSION

Successful endoscopic removal of tumors sinonasal vascular origin can be achieved by utilizing the LigaSure™ vessel sealing device, without the need for preoperative tumor embolization.

## AUTHOR CONTRIBUTIONS

**Hong Jin Kim:** Writing – original draft. **Ye Hwan Lee:** Data curation. **Seung Hoon Lee:** Supervision. **Min Young Seo:** Conceptualization; writing – original draft; writing – review and editing.

## FUNDING INFORMATION

This study was funded by Korea University Ansan Hospital (O2310621).

## CONFLICT OF INTEREST STATEMENT

None.

## CONSENT

Written informed consent was obtained from the patient to publish this report in accordance with the journal's patient consent policy.

## Data Availability

The data that support the findings of this study are not openly available due to reasons of sensitivity and are available from the corresponding author upon reasonable request. Data are located in controlled access data storage at Korea University College of Medicine.
